# Anti- IgE treatment in asthma: is atopy essential?

**DOI:** 10.1186/1939-4551-8-S1-A150

**Published:** 2015-04-08

**Authors:** Mustafa Gulec, Ali Selcuk, Ozgur Kartal, Fevzi Demirel, Sait Yesillik, Abdullah Baysan, Ugur Musabak, Osman Sener

**Affiliations:** 1 Gülhane Military Medical Academy and Medical School, Turkey

## Background

Omalizumab is a biologic molecule which is used on severe allergic asthma patients. Omalizumab, which shows effect by binding to free IgE molecule in circulation, is reported to be effective in nonallergic asthma patients in some case reports.

## Methods

Case 1, a fifty one-year old woman who has been treated for 11 years diagnosis of asthma, went to emergency service four times last year in spite of taking high dose inhaler corticosteroid. In her physical examination widespread rhonchi was oscultated. FEV1: %76, total IgE: 897 IU/mL inhalant skin prick tests and mites spesific IgE were negative. Visual Analog Score(VAS) was 2, asthma symptom score (ASS) was determined as 6. Omalizumab was started 450mg/month as diagnosis of nonallergic asthma. One week after the first injection of omalizumab, patient's complaints got better. The patient is taking omalizumab for ten months and VAS is 8, ASS is 2, can use salbutamol if necessary.

Case 2, a sixty nine-year old woman patient has hypertension, epilepsy, anxiety disorder as well as 12 years of asthma. She consulted the emergency countless times and stayed in hospital twice last year. The patient is still using high dose of inhaler corticosteroid and using oral corticosteroid constantly. FEV1: %73, inhalant skin prick tests were negative. Total IgE: 116 IU/mL mite and mold spesific IgE were negative. At the begginning, the patient whose VAS 3, ASS 8, is taking 300 mg of Omalizumab every month. The patient's symptoms got better after the second dose of treatment and the VAS was 8, ASS was 3 in the 9th month of omalizumab. The patient is still using one dose of budesonid/formoterol and the other disease is under control.

In both of the cases, there wasn't any emergency consult or hospitilization.

## Results

The text does not involve results.

## Conclusions

The clinical efficiency of omalizumab on normal-severe allergic asthmas is showed by a lot of studies. GINA(Global Initiative of Asthma) is said to be a treatment choice for patients who are sensitive of perennial allergens. Although IgE levels were high in both of our cases, not only skin prick tests but also they were patients whose perennial allergenic spesific IgE was negative. Recently studies show that there is no difference in inflammatory cytokines releasing or expression of high affinity Ig E receptor allergic or non-allergic asthma. We know that effect of omalizumab is via free IgE molecule in circulation. In this effect, role of atopy is arguable.

## Consent

Written informed consent was obtained from the patient for publication of this abstract and any accompanying images. A copy of the written consent is available for review by the Editor of this journal.

